# The Bitter Aftertaste of Kratom: A Toxicologic and Neurologic Challenge

**DOI:** 10.7759/cureus.111809

**Published:** 2026-06-30

**Authors:** Aditya Lal Vallath, Rashmitha Pippari, Ratan Yadav, Michelle Bass, Mario Deyulis

**Affiliations:** 1 Emergency Medicine, Conemaugh Memorial Medical Center, Johnstown, USA; 2 Internal Medicine, Conemaugh Memorial Medical Center, Johnstown, USA; 3 School of Medicine, Universidad El Bosque, Bogota, COL

**Keywords:** critical care, drug addiction, emergency medicine, kratoum, substance overdose

## Abstract

Kratom (*Mitragyna speciosa*) is an increasingly used botanical substance with opioid-like properties. Products containing 7-hydroxymitragynine (7-OH), a potent μ-opioid receptor agonist, have been associated with severe toxicity, though clinical data remain limited.

We report the case of a woman with polysubstance use disorder who presented following intentional ingestion of a large quantity of 7-OH. She developed profound central nervous system depression, marked QTc prolongation, and prolonged encephalopathy requiring intensive care admission and airway protection. Her hospital course was complicated by persistent neuropsychiatric symptoms, prompting extensive neurologic and psychiatric evaluation, including electroencephalography and cerebrospinal fluid analysis. Findings were consistent with a multifactorial encephalopathy related to toxic exposure, withdrawal, and underlying psychiatric illness, with possible infectious contribution. She improved with supportive care, cardiac monitoring, antimicrobial therapy, and psychiatric intervention, and was ultimately transferred to inpatient psychiatric care and substance-use rehabilitation.

This case highlights the potential for severe and prolonged neurocardiac and neuropsychiatric toxicity following 7-OH ingestion and underscores the importance of clinical awareness of kratom-related complications, particularly in patients with polysubstance use and psychiatric comorbidities.

## Introduction

Kratom, a botanical product derived from the *Mitragyna speciosa* tree native to Southeast Asia, has seen a surge in unregulated use within the United States as a purported natural remedy for analgesia, mood enhancement, and opioid withdrawal management. Despite its marketing as a safe alternative to traditional opioids, the plant contains a complex profile of bioactive alkaloids, most notably mitragynine and its more potent derivative, 7-hydroxymitragynine (7-OH). While mitragynine is the primary alkaloid by volume, 7-OH exhibits significantly higher affinity for mu-opioid receptors and is largely responsible for the plant's opioid-like physiological effects. The proliferation of concentrated extracts and synthetic formulations has introduced new risks for unpredictable toxicity [1-2].

Growing evidence links kratom exposure with severe adverse outcomes, including central nervous system (CNS) depression, psychosis, seizures, hepatotoxicity, and cardiac conduction delays such as QT interval prolongation [1]. We report a case of marked QTc prolongation and a protracted state of encephalopathy following intentional ingestion of a large quantity of 7-OH. The clinical picture was complicated by polysubstance use disorder, psychiatric illness, and evaluation for possible Lyme neuroborreliosis. This highlights the diagnostic and management challenges associated with severe toxicity in the setting of multiple potential contributors to prolonged neuropsychiatric dysfunction.

## Case presentation

A 30-year-old woman with a past medical history significant for polysubstance use disorder, Crohn’s disease, Hashimoto thyroiditis with hypothyroidism, essential hypertension, endometriosis, anxiety, and chronic low back pain with left-sided sciatica was brought to the emergency department (ED) by emergency medical services (EMS) following an intentional overdose in a suicide attempt.

**Figure 1:**
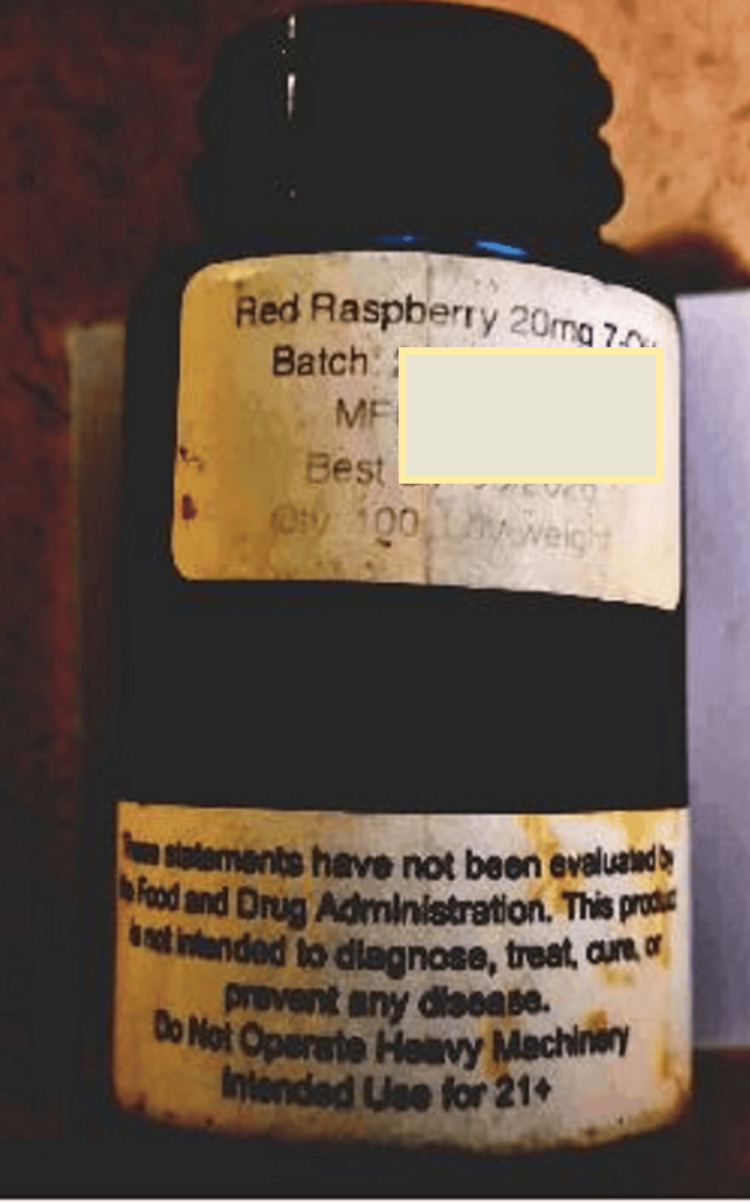
Bottle of “Red Raspberry 20 mg 7-OH” kratom ingested by the patient, with label warnings and the U.S. Food and Drug Administration (FDA) disclaimer.

According to EMS, the patient ingested approximately twenty 25-mg tablets of 7-OH kratom (Figure 1) approximately one hour before arrival. During transport, she developed nausea and progressive alteration in mental status. She was treated prehospital with ondansetron 4 mg intravenously for nausea and naloxone 0.5 mg intravenously for suspected opioid toxicity, with minimal improvement in mental status.

**Figure 2:**
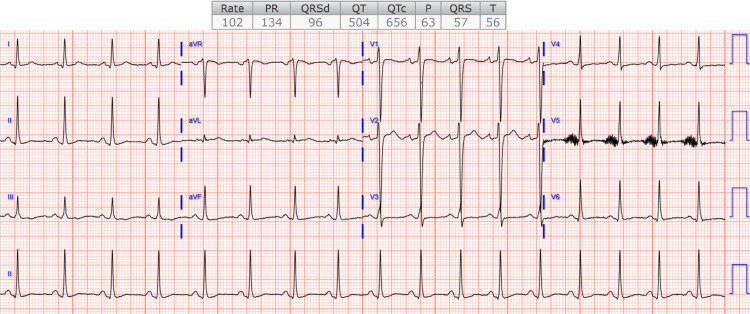
Twelve-lead electrocardiogram (ECG) on emergency department (ED) arrival. The ECG demonstrates sinus tachycardia at a rate of 102 bpm, with a markedly prolonged corrected QT (QTc) interval of 656 ms. QRS duration is normal. No ST-segment elevation or depression is noted.

On arrival to the ED, the patient was somnolent and minimally responsive to painful stimuli. She was initially protecting her airway. Initial vital signs demonstrated a temperature of 36.8 °C, heart rate of 84 beats per minute, blood pressure of 103/63 mmHg, respiratory rate of 18 breaths per minute, and oxygen saturation of 100% on room air. Cardiovascular and respiratory examinations were unremarkable. Neurologic examination revealed no focal deficits; however, she exhibited markedly depressed responsiveness with psychomotor retardation. A 12-lead electrocardiogram obtained on arrival demonstrated sinus rhythm with a markedly prolonged corrected QT interval (QTc) of 656 ms (Figure 2). 

Initial laboratory evaluation revealed a normal complete blood count and basic metabolic panel, including preserved renal function. Liver function testing demonstrated mild transaminitis with an aspartate aminotransferase (AST) of 50 U/L and alanine aminotransferase (ALT) of 67 U/L. Urine drug screening was positive for opioids, cannabinoids, and tricyclic antidepressants. Serum acetaminophen and salicylate levels were undetectable, and serum ethanol was negative. As part of the ED evaluation, head CT demonstrated no acute intracranial abnormality, and computed tomography (CT) of the abdomen and pelvis demonstrated hepatic enhancement concerning for acute hepatocellular dysfunction.

Poison Control was contacted early in the ED course, and the patient’s ingestion history, electrocardiographic findings, laboratory results, and clinical status were reviewed. Based on this assessment, Poison Control recommended intravenous magnesium sulfate for QTc prolongation and repeat naloxone dosing. In accordance with these recommendations, the patient received magnesium sulfate 2 g intravenously and multiple doses of naloxone (0.4-1 mg IV), without meaningful improvement in mental status. Her level of consciousness continued to decline, and she became responsive only to painful stimuli, raising concern for loss of airway protection and aspiration risk. The patient subsequently underwent rapid sequence intubation for airway protection using etomidate (10 mg IV) and rocuronium (50 mg IV). Following successful endotracheal intubation, she was initiated on a continuous midazolam infusion for sedation, selected due to its minimal effect on QT interval prolongation. She was admitted to the intensive care unit (ICU) for close monitoring and management of suspected kratom toxicity.

**Figure 3:**
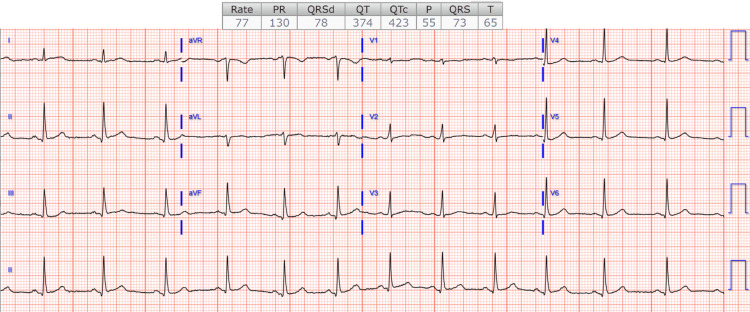
Improvement in ECG findings at 96 hours. The ECG demonstrates a normal sinus rhythm at a rate of 77 bpm, with a normal QTc interval of 423 ms. QRS duration is normal. No ST-segment elevation or depression is noted. ECG, electrocardiogram; QTc, corrected QT; QRS, QRS complex; bpm, beats per minute

During her ICU course, serial electrocardiograms demonstrated gradual improvement of the QTc interval, which normalized to approximately 440 ms over the subsequent 96 hours (Figure 3). Despite improvement in cardiac conduction, the patient remained encephalopathic beyond the anticipated period of acute intoxication based on available pharmacologic data. On the first ICU day, she self-extubated, was placed on a non-rebreather mask, remained hemodynamically stable, followed commands, and did not require re-intubation.

Further evaluation for metabolic and infectious causes of altered mental status was pursued. Electroencephalography demonstrated generalized background slowing without epileptiform discharges, consistent with diffuse encephalopathy. Lyme serologic testing returned positive for IgM antibodies. Lumbar puncture revealed mild lymphocytic pleocytosis with modest protein elevation and normal glucose; cerebrospinal fluid Lyme polymerase chain reaction testing was negative. Neurology assessed the findings as suggestive of possible neuroborreliosis in the appropriate clinical context and recommended antimicrobial therapy. The patient was treated initially with intravenous ceftriaxone and subsequently transitioned to oral doxycycline to complete a total 21-day course. Neurology concluded that her encephalopathy was likely multifactorial, related to polysubstance ingestion, withdrawal, underlying psychiatric disease, and possible infection, and cleared her for psychiatric care.

Following extubation, the patient exhibited waxing and waning mentation, disorganized behavior, insomnia, and persistent suicidal ideation, necessitating repeated psychiatric evaluations. She required continuous one-to-one observation and intermittent restraints for safety. Psychiatric management initially included orally disintegrating olanzapine; however, due to limited clinical response and concern for QTc prolongation, therapy was transitioned to scheduled haloperidol with additional intravenous doses as needed for agitation. Daily electrocardiographic monitoring and electrolyte repletion were maintained throughout treatment.

Based on her clinical course, toxicologic findings, and psychiatric assessment, her presentation was attributed to a combination of acute kratom toxicity, polysubstance withdrawal, and underlying psychiatric illness. Once medically stabilized, she was transferred to the inpatient medical floor and subsequently admitted to an inpatient psychiatric facility, where she completed further treatment before discharge to a structured substance-use rehabilitation program.

## Discussion

Kratom, scientifically known as *Mitragyna speciosa Korth*, is a tropical evergreen tree in the Rubiaceae family indigenous to the warm, humid climates of Southeast Asia, including Thailand, Malaysia, Myanmar, Cambodia, Indonesia, and Papua New Guinea. The tree can grow up to 50 feet tall, with a spread of more than 15 feet, and is distinguished by its dark green, glossy leaves. Different varieties of kratom contain varying levels of active chemical compounds, primarily alkaloids, which are responsible for its diverse effects [1]. For centuries, kratom has been deeply embedded in the traditional medicinal practices and cultural life of indigenous populations in Southeast Asia. Its use has historically served both functional and therapeutic purposes. In countries such as Thailand and Malaysia, laborers commonly chewed kratom leaves to combat fatigue, enhance stamina, and maintain alertness during long hours of manual labor. This practice associated kratom with physical endurance and resilience in agrarian communities. Beyond its stimulant properties, kratom was traditionally employed as a versatile remedy for a range of ailments, including the treatment of musculoskeletal pain, coughs, diarrhea, intestinal infections, parasitic infestations, and topical use for wound care and as a local anesthetic. Notably, kratom served as an alternative to opium, particularly during periods of scarcity or for individuals attempting to manage opioid withdrawal symptoms, a practice documented in Malaysia as early as 1836 and later in Thailand throughout the 19th century [2].

In recent years, particularly in southern Thailand, a new method of kratom consumption has emerged: homemade cocktails known as *4x100*, which combine kratom leaves with a caffeinated soft drink and cough syrup containing codeine or diphenhydramine, and in some cases, additional psychoactive substances such as anxiolytics, antidepressants, or analgesics. These mixtures are consumed for their alcohol-like intoxicating effects. This shift marks a significant evolution in kratom use from traditional botanical preparations to forms of polysubstance use aimed at enhanced psychoactive experiences [3-4].

The historical use of kratom as an opium substitute, combined with its resurgence during the U.S. opioid crisis, particularly among individuals seeking to manage withdrawal symptoms, underscores a transnational continuity in the perception of kratom as a tool for mitigating opioid dependence. Its current use in the U.S. opioid crisis reflects both its pharmacological appeal and its cultural legacy as a plant with opioid-like effects [1].

The practice of combining kratom with other psychoactive agents, whether for recreational, self-medicating, or social purposes, has parallels with the patterns of complex overdose scenarios now reflected in the modern U.S. drug landscape. This historical and regional trajectory highlights that the risks associated with polysubstance use involving kratom are not new, but rather have evolved and intensified with the proliferation of potent synthetic drugs [5]. Fentanyl and its analogs were the most frequently co-detected substances among kratom-positive decedents, identified in 65.1% of cases, followed by heroin, benzodiazepines, and prescription opioids. This pattern indicates that kratom is often used concomitantly with other substances, particularly potent opioids, rather than as a primary agent.

Notably, only a small number of fatalities - seven in a Centers for Disease Control and Prevention (CDC) investigation and two in a report by the National Institute on Drug Abuse (NIDA) - have been conclusively attributed to kratom exposure alone. Independent reviews commissioned by the American Kratom Association further corroborate these findings, concluding that most kratom-associated deaths were the result of polydrug use or contamination with adulterants [6]. Approximately 80% of decedents in kratom-involved deaths had documented histories of substance use disorders, and nearly 90% were not receiving treatment for pain at the time of death. This data suggests that kratom is frequently consumed by individuals with pre-existing vulnerabilities, as part of a broader, high-risk polysubstance use profile, a pattern directly relevant to the present case [7].

Kratom preparations contain several phytochemicals in varying ratios , rendering their proper pharmacological evaluation difficult. Human clinical studies are scarce. The two principal alkaloids, *mitragynine* and *7-hydroxymitragynine*, have been identified as the primary contributors to kratom’s psychoactive and analgesic effects. Pharmacologically, both mitragynine and 7-OH function as full agonists at the μ-opioid receptor (MOR), a key receptor subtype involved in analgesia, reward, and addiction pathways. 7-OH exhibits higher affinity and potency at MOR than mitragynine, despite being present in lower concentrations. These effects are reversibly antagonized by naloxone, indicating a mechanistic overlap with classical opioids [8]. In addition to MOR activation, mitragynine is an agonist at 5-hydroxytryptamine 2A (5-HT2A) receptors and α2-adrenergic receptors and modulates neuronal Ca²⁺ channels. These effects likely impact neurotransmitter release, contributing to its modulatory effects on mood and perception, as well as its stimulant and analgesic properties.

The onset of action following oral ingestion of 2 to 5 g of dried kratom leaves is typically within 10 minutes, with stimulant-like effects lasting 1 to 1.5 hours. These effects include increased alertness, sociability, physical endurance, and occasionally enhanced libido. Somatic signs such as mild facial flushing and slight pupillary constriction may occur. At higher doses (10 to 25 grams), kratom produces CNS depressant effects. Users typically report initial nausea, dizziness, sweating, and dysphoria, followed by calmness, euphoria, and a dreamlike state lasting up to six hours. Miosis is more consistently observed at these sedative doses [9].

The metabolic fate of mitragynine in humans involves several pathways: hydrolysis of the side-chain ester, O-demethylation of methoxy groups, oxidative/reductive biotransformation, and subsequent phase II conjugation via glucuronidation and sulfation. These metabolic processes contribute to the variability in individual responses and may influence the duration and intensity of pharmacological effects. It is also a potent inducer of Cytochrome P-450 and inhibitor of CYP2D6 and CYP3A [10]. Given its complex receptor pharmacology and variable alkaloid profile, kratom exhibits a unique biphasic dose-response curve, with stimulant effects at lower doses and sedative, opioid-like effects at higher doses [11].

The potential for dependence and withdrawal associated with long-term kratom use presents a significant public health concern, particularly as its consumption becomes more widespread outside traditional Southeast Asian contexts. Evidence from observational studies in Malaysia indicates that habitual kratom users may maintain daily use over decades, with one cohort reporting average usage durations of approximately 18.6 years. The withdrawal symptoms described in these cases, such as hostility, aggression, emotional lability, rhinorrhea, myalgia, and involuntary limb movements, mirror those seen in opioid withdrawal syndromes, albeit with some reports suggesting a less intense symptom profile [12-14]. Nonetheless, the severity of kratom withdrawal remains a point of debate. While certain reviews argue that kratom's withdrawal symptoms are milder compared to those induced by classical opioids, others emphasize that the syndrome can still be clinically significant and impairing, particularly in chronic users. This ambiguity reflects the broader variability in individual response to kratom’s alkaloid composition, dosage, and duration of use [15].

Survey data from the United States further underscore the addiction potential of kratom, with over one-quarter (25.5%) of users reporting symptoms consistent with a substance use disorder. Such findings suggest that kratom may not be as benign as often assumed, especially in nontraditional settings where use may be unregulated and combined with other substances [16]. The high relapse rates following cessation, ranging from 78% to 89% at three months, further highlight the challenge of maintaining abstinence in dependent individuals [17]. These rates are comparable to those observed in opioid use disorder, reinforcing the argument that treatment approaches for severe kratom dependence may need to mirror those used for opioids, including both pharmacological and behavioral interventions [18].

Accurately estimating kratom use in the United States is difficult due to its unregulated status and the limitations of national data collection. According to the 2021 National Survey on Drug Use and Health by SAMHSA, approximately 1.7 million Americans aged 12 and older reported kratom use. However, other sources present significantly higher figures. Estimates from 2020 suggest that up to 15 million Americans may have used kratom, while the American Kratom Association claims that 10-15 million people have tried it, with around 5 million current regular users. Independent studies also suggest that national surveys may underestimate the prevalence, placing lifetime use somewhere between 1 and 6 million [19].

This wide range of estimates from 1.7 to 15 million users reveals a significant gap in national surveillance efforts and highlights the challenges posed by kratom’s legal and regulatory ambiguity. The lack of reliable prevalence data hampers public health planning, making it difficult for authorities to evaluate potential risks, allocate resources, and develop informed regulatory strategies. Until kratom use is systematically monitored, policymaking will remain reactive rather than preventive, undermining efforts to manage both the therapeutic and harmful dimensions of its growing use [20].

Commercially available kratom products span a broad range from tablets, teas, capsules, and tinctures to newer synthetic forms with higher potencies [1]. Current scientific consensus indicates that 7-OH is present in kratom leaves only in trace amounts, likely as a degradation byproduct rather than a primary natural constituent. Despite its minimal presence in the raw plant material, isolated or synthetically produced 7-OH exhibits significantly greater pharmacological potency. It is estimated to be 13 times more potent than mitragynine and up to 46 times stronger than morphine at the MOR. Moreover, synthetic analogs of 7-OH can exhibit up to 13-fold higher potency compared to the naturally occurring trace amounts, raising concerns about their potential for misuse and adverse effects [21-22].

In the present case, it was noted that the patient had consumed a synthetic version of kratom, which most likely accounts for the severity of her initial CNS depression and QTc prolongation. The patient’s encephalopathy persisted beyond the expected pharmacokinetic window of 7-OH, prompting evaluation for additional contributors. Positive IgM antibodies to Lyme disease were identified, although CSF analysis was negative, highlighting a diagnostic conundrum of possible Neuroborreliosis.

Neuroborreliosis arises when Borrelia bacteria, transmitted through tick bites, spread from the initial site of infection via the bloodstream and lymphatic system to reach the central and peripheral nervous systems. The bacteria infiltrate the CNS by crossing the blood-brain or blood-spinal cord barriers through paracellular, transcellular, or *Trojan horse* mechanisms. Once inside the nervous system, Borrelia induces a multifocal inflammatory response by activating immune cells such as macrophages and monocytes. These cells release proinflammatory cytokines (e.g., interleukin-6 (IL-6), tumor necrosis factor-α (TNF-α)) and chemokines (e.g., CXCL13), promoting neuroinflammation, oxidative stress, and apoptotic signaling pathways that ultimately damage neurons. Additionally, the bacteria exhibit both direct and indirect neurotoxic effects, degrade tight junction proteins in the CNS barriers, and can trigger autoimmune responses through molecular mimicry, all of which contribute to neuronal injury [23-27]. Clinical symptoms in early neuroborreliosis include peripheral neuropathy, cranial neuritis (commonly facial palsy), and meningitis, while late-stage symptoms can progress to chronic meningitis, polyneuropathy, encephalitis, and myelitis [28].

In this case, the positive IgM serology in the setting of persistent encephalopathy and a history of chronic low back radiculopathy raised the possibility of neuroborreliosis as a contributing factor. However, the negative CSF polymerase chain reaction (PCR) substantially weakens the case for active CNS infection, and isolated IgM positivity can reflect prior exposure rather than active disease. Neuroborreliosis should therefore be considered a possible, but unconfirmed, contributor to this patient’s neuropsychiatric course. A 21-day antibiotic course (IV ceftriaxone followed by oral doxycycline) was consistent with guideline-based management for suspected neuroborreliosis, and the patient improved with this regimen [29].

Taken together, the clinical evidence most strongly supports acute synthetic 7-OH toxicity as the primary driver of this patient’s initial CNS depression and QTc prolongation. The concurrent positive urine drug screen for opioids, cannabinoids, and tricyclic antidepressants indicates meaningful polysubstance exposure, which likely compounded the acute toxidrome and contributed substantially to the protracted neuropsychiatric course through withdrawal. Underlying psychiatric illness represents an additional contributor that independently shaped her presentation. Lyme neuroborreliosis, while empirically treated, remains diagnostically unconfirmed and should be interpreted as a lower-confidence contributor whose precise role cannot be disentangled from the overlapping toxic and withdrawal states.

## Conclusions

This case illustrates the potential for severe neurocardiac and neuropsychiatric toxicity following intentional ingestion of synthetic 7-OH, compounded by polysubstance exposure, withdrawal, and underlying psychiatric illness. The co-diagnosis of neuroborreliosis may have played a contributory role in the patient's neuropsychiatric symptoms, emphasizing the importance of thorough neurologic and infectious workups in atypical presentations. 

Kratom, though often marketed as a natural or safe alternative for mood and pain management, can contribute to significant psychiatric and physiological harm and its accessibility remains a pressing concern. Despite being unscheduled at the federal level, its psychoactive components are banned in several U.S. states and locally regulated in others. The patchwork of state-level regulations, including the adoption of the Kratom Consumer Protection Act (KCPA) in multiple jurisdictions, reflects both growing awareness and regulatory uncertainty. Greater regulatory oversight, standardized product testing, and heightened clinical vigilance are essential to address gaps in safety and establish public health protections to identify and treat individuals at risk for kratom misuse. 
